# A Recyclable Cu-MOF-74 Catalyst for the Ligand-Free O-Arylation Reaction of 4-Nitrobenzaldehyde and Phenol

**DOI:** 10.3390/nano7060149

**Published:** 2017-06-16

**Authors:** Pedro Leo, Gisela Orcajo, David Briones, Guillermo Calleja, Manuel Sánchez-Sánchez, Fernando Martínez

**Affiliations:** 1 Department of Chemical and Energy Technology, ESCET. Rey Juan Carlos University, C/Tulipan s/n, 28933 Mostoles, Spain; pedro.leo@urjc.es (P.L.); gisela.orcajo@urjc.es (G.O.); david.briones@urjc.es (D.B.); guillermo.calleja@urjc.es (G.C.); 2 Instituto de Catálisis y Petroleoquímica, ICP-CSIC. C/Marie Curie 2, 28049 Madrid, Spain; manuel.sanchez@icp.csic.es

**Keywords:** MOF, catalyst, recyclable Cu-MOF-74, ligand-free, O-arylation reaction, 4-nitrobenzaldehyde, phenol, 4-formyldiphenyl ether

## Abstract

The activity and recyclability of Cu-MOF-74 as a catalyst was studied for the ligand-free C–O cross-coupling reaction of 4-nitrobenzaldehyde (NB) with phenol (Ph) to form 4-formyldiphenyl ether (FDE). Cu-MOF-74 is characterized by having unsaturated copper sites in a highly porous metal-organic framework. The influence of solvent, reaction temperature, NB/Ph ratio, catalyst concentration, and basic agent (type and concentration) were evaluated. High conversions were achieved at 120 °C, 5 mol % of catalyst, NB/Ph ratio of 1:2, DMF as solvent, and 1 equivalent of K_2_CO_3_ base. The activity of Cu-MOF-74 material was higher than other ligand-free copper catalytic systems tested in this study. This catalyst was easily separated and reused in five successive runs, achieving a remarkable performance without significant porous framework degradation. The leaching of copper species in the reaction medium was negligible. The O-arylation between NB and Ph took place only in the presence of Cu-MOF-74 material, being negligible without the solid catalyst. The catalytic advantages of using nanostructured Cu-MOF-74 catalyst were also proven.

## 1. Introduction

Diaryl ethers are very valuable organic compounds in the synthesis of biologically natural products and in the pharmacological and polymer fields [1,2]. The traditional synthesis is based on the Ullmann cross-coupling reaction of phenols with aryl halides, catalyzed by copper salts [3]. However, homogeneous copper-mediated coupling reactions are limited by the large amount of catalyst needed and harsh reaction conditions [4,5,6,7]. The high concentration of the catalyst is an important environmental drawback, as large quantities of hazardous copper-based waste are produced. Additionally, it has been reported that additives or ligands are needed for promoting the ether formation at milder reaction conditions [8,9]. The synthesis of diaryl ethers with palladium as a catalyst has been also reported [10], but the need of expensive palladium amounts and complex ligands limit its feasibility. The development of copper heterogeneous catalysts for Ullmann C–O cross-coupling reactions is therefore a motivating challenge for a more sustainable process, as they could be easily recoverable and reusable and probably not requiring assisting ligands. 

Up to date, very few works have been addressed to this issue [11,12]. Magnetic CuFe_2_O_4_ nanoparticles showed remarkable performances for the C–O cross reaction of phenols with aryl halides, although acetylacetone as ligand was necessary [13]. More recently, metal-organic framework (MOF) materials have been evaluated. MOF materials are crystalline solids with surface areas surpassing activated carbons and zeolites, composed by metal nodes connected by organic linkers through strong chemical bonds [14] to lead to high order and uniform porous networks for chemical-, size- and shape-selective catalysis [15,16,17,18,19]. Phan and co-workers have paid attention on ligand-free copper-catalyzed coupling reactions of phenols with aryl iodides or nitroarenes [20] using MOF-199 and Cu_2_(BDC)_2_(DABCO) (BDC = 1,4-benzene dicarboxylate and DABCO = 1,4-diazabicyclo[2.2.2]octane), respectively. Maity and coworkers have also investigated the O-arylation of aryl alcohols with aryl bromides using a Zn-based isoreticular metal organic framework (IRMOF-3) in which copper(II) was anchored following a post-synthetic modification method [21]. The most stimulating features of MOF materials for catalytic applications are their high surface area, tunable pore size, scaffold flexibility, and diversity of structural functional sites like the accessible metallic centers [22].

In this work, the Cu-MOF-74 material, characterized by exhibiting unsaturated copper sites pointing to the pore channels, was studied as a catalyst of the free-ligand O-arylation reaction of phenol with 4-nitrobenzladehyde. This Cu-MOF-74 material was previously tested in the acylation of anisole with phenol, exhibiting remarkable catalytic activity and structural stability [23]. The influence of different reaction variables such as temperature, type of solvent, catalyst concentration and type and concentration of base in the absence of assisting ligands was evaluated in order to determine the best operation conditions for the synthesis of diaryl ethers. The recyclability of the catalyst was also studied along several successive catalytic runs, checking its resistance to activity loss. Finally, this MOF material was compared to the nanostructured homologue Cu-MOF-74 in order to study the influence of the catalyst’s crystal size over its catalytic performance. 

## 2. Results and Discussion

### 2.1. Characterization of Cu-MOF-74

**Figure 1:**
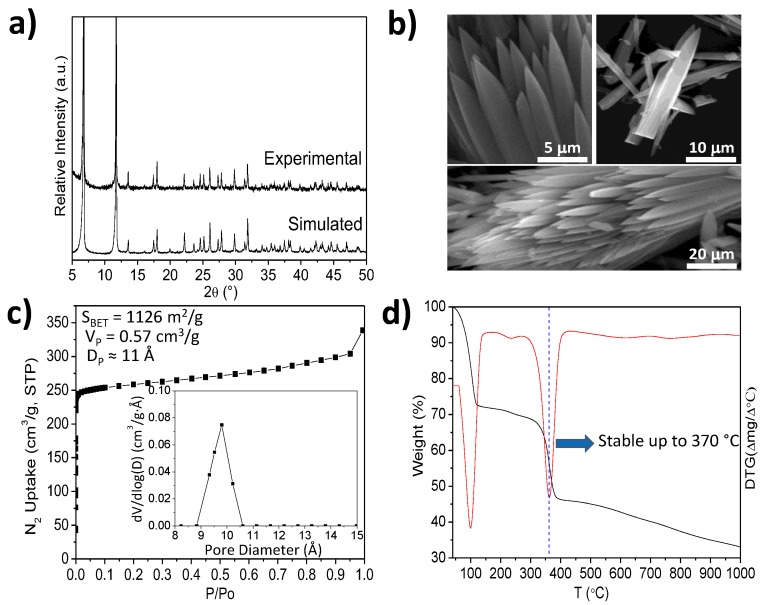
Catalyst characterization of Cu-MOF-74: (**a**) PXRD patterns; (**b**) SEM images; (**c**) nitrogen adsorption–desorption isotherms at −196 °C with an inset showing the micropore size distribution curve as generated by NL-DFT method, and (**d**) TGA under inert atmosphere.

Cu-MOF74 was synthesized by the solvothermal method previously described [24] with a yield of 74%. Conventional techniques were used for the physico-chemical characterization of Cu-MOF-74 material as shown in Figure 1. Powder X-ray diffraction (PXRD) pattern revealed the typical reflections of the MOF-74/CPO-27 phase [25], discarding the presence of secondary crystalline phases (Figure 1a). SEM micrographs also revealed the expected large needle-shaped crystals that have been previously reported for this Cu-MOF-74 material [24] (Figure 1b). Both PXRD patterns and SEM pictures confirmed the crystallinity of the Cu-MOF-74 material. Elemental analysis by using ICP-OES indicated a copper loading of 6.1 mmol/g, which is close to the theoretical content from the molecular structure (6.23 mmol/g). The porosity of the material was measured by nitrogen adsorption at −196 °C (Figure 1c). The isotherms revealed a microporous material with a BET specific surface area around 1100 m^2^/g, a pore volume of 0.57 cm^3^/g, and an average pore diameter of ca. 10.1 Å. Thermogravimetric analysis (TGA) under N_2_ atmosphere evidenced a first weight loss at ca. 100 °C corresponding to the solvent removal from the porous framework as well as a high thermal stability until 370 °C, when the decomposition of the organic ligand takes place (Figure 1d).

### 2.2. Catalytic Study

#### 2.2.1. Influence of Temperature

**Figure 2:**
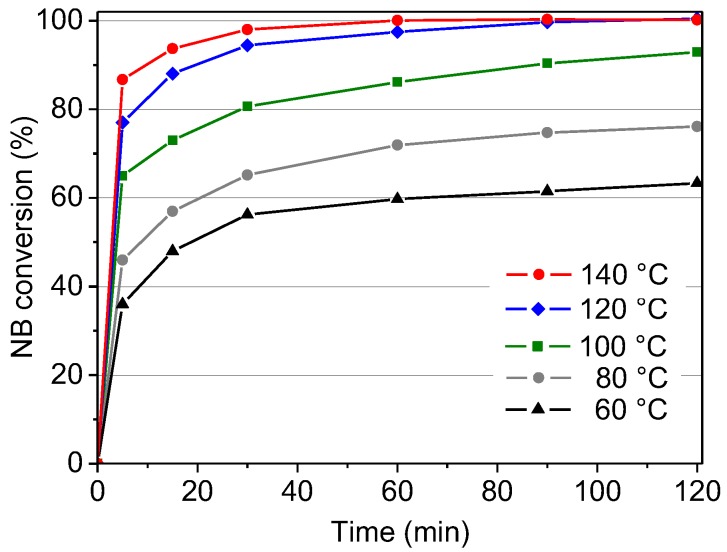
Kinetics of 4-nitrobenzaldehyde (NB) conversion catalyzed by Cu-MOF-74 at different temperatures

The influence of the temperature on the O-arylation cross-coupling reaction of 4-nitrobenzaldehyde (NB) and phenol (Ph) to form 4-formyldiphenyl ether was evaluated at 60, 80, 100, 120, and 140 °C (Figure 2). These reactions were carried out in *N*,*N*-dimethylformamide (DMF) as solvent, with an NB/Ph molar ratio of 1/2, 2 equivalents of K_2_CO_3_ as a base, and 5 mol % of catalyst, conditions similar to those previously published [20]. A remarkable 88% conversion of NB was achieved after 6 min and 100% conversion after 1 h for the highest reaction temperature (140 °C). The decrease of temperature reduced the catalytic activity of Cu-MOF-74, being less significant in the range of 120–140 °C. At the lowest tested temperature (60 °C), the NB conversion after 2 h was ca. 60%. At the reaction temperature of 100 °C, the Cu-MOF-74 showed a catalytic activity similar to another Cu-based MOF material (Cu_2_(BDC)_2_(DABCO)) reported in the literature [20]. However, Cu_2_(BDC)_2_(DABCO) evidenced a stronger loss of activity at lower reaction temperatures as compared to Cu-MOF-74. This behavior is attributed to the average pore diameter and diffusional constraints of both materials when the temperature is decreased. The pore size diameter of Cu-MOF-74 (10.1 Å) is larger than that reported for Cu_2_(BDC)_2_(DABCO) (6.4 Å), a difference that enables an easier diffusion of reactants within the porous structure of Cu-MOF-74, favoring its catalytic activity. On the other hand, the Cu_2_(BDC)_2_(DABCO) material was not tested at temperatures above 100 °C [20], whereas the catalytic performance of Cu-MOF 74 is significantly enhanced. Therefore, the Cu-MOF-74 is active at mild reaction temperatures (60 °C) and becomes extremely active at 120 °C, giving an NB conversion close to 80% in few minutes.

**Figure 3:**
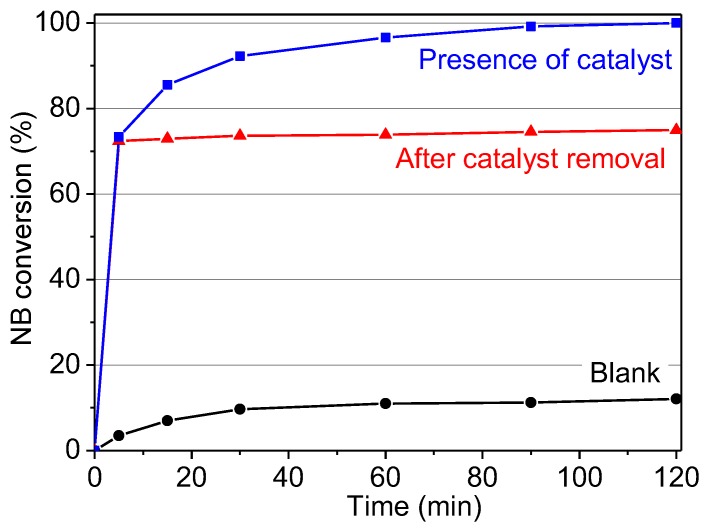
NB conversion in the absence of catalyst (blank), in the presence of Cu-MOF-74 catalyst for 120 min and in the presence of Cu-MOF-74 for 5 min and subsequent removal.

In order to evaluate the contribution of homogeneous catalysis by possible leached copper species from Cu-MOF-74, an additional catalytic run at 120 °C was carried out, removing the solid catalyst from the mixture by hot filtration after 5 min of reaction. The reaction mixture was further maintained for 2 h under these conditions. Figure 3 shows the NB conversion profiles with the solid Cu-MOF-74 catalyst present all the time (called “presence of catalyst”) and the so-called “after catalyst removal” with the Cu-MOF-74 catalyst removed after 5 min. The active role of the heterogeneous Cu-MOF-74 catalyst in the O-arylation cross-coupling reaction is clearly proven when comparing both profiles. No further significant conversion of NB was detected once the solid catalyst was removed from the reaction mixture, which demonstrates a negligible contribution of homogeneous catalysis by plausible copper leaching. In fact, dissolved copper species were not detected in the reaction mixture by ICP-OES analyses.

#### 2.2.2. Influence of 4-Nitrobenzaldehyde/Phenol Molar Ratio

**Figure 4:**
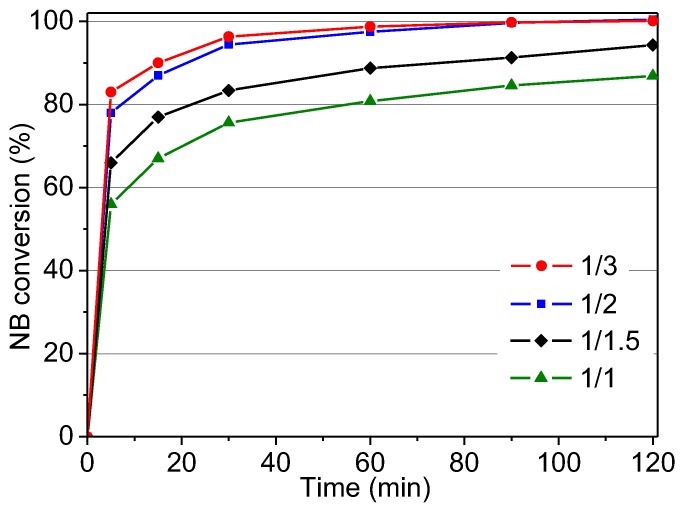
NB conversion vs. reaction time given by Cu-MOF-74 catalyst at different NB/Ph ratios.

The influence of the reactants ratio in the cross-coupling reaction was also investigated, testing the NB/Ph molar ratios of 1/1, 1/1.5, 1/2, and 1/3 (Figure 4). These reactions were carried out at 120 °C with DMF as solvent and 2 equivalents of K_2_CO_3_ as base in the presence of 5 mol % of Cu-MOF-74 catalyst. A significant effect of the NB/Ph ratio on the conversion is clearly observed. The increase of phenol proportion until NB/Ph = 1/2 produced a significant enhancement of the NB conversion. It must be noted that similar results were reported using other copper-based MOF materials, like Cu_2_(BDC)_2_(DABCO), being the best NB/Ph molar ratio 1/1.5 [20]. This result can be explained by the role of phenol as a ligand in the catalytic mechanism, which accelerates the coupling with aryl halides, as observed in homogeneous copper catalytic systems [26]. In the case of heterogeneous Cu-MOF materials, the benefit of using phenol in excess is also probably related to the primary interaction of phenolates with available copper sites followed by coupling with NB, instead of complexation of NB as an electrophilic reactant and later coupling with phenolate as a nucleophilic agent.

#### 2.2.3. Influence of Catalyst Concentration

**Figure 5:**
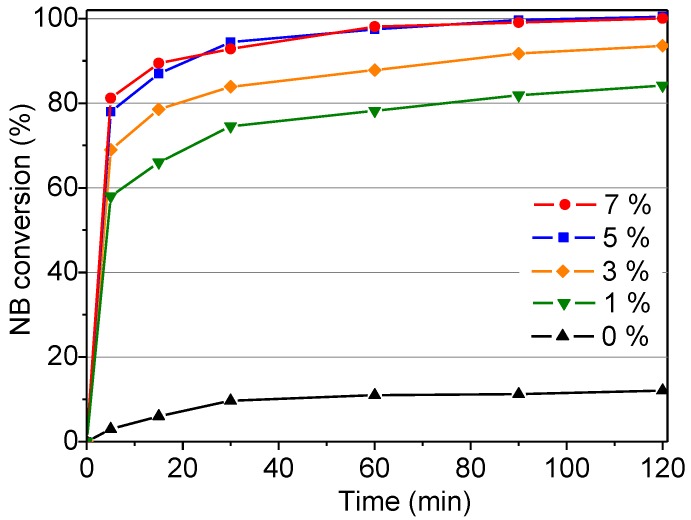
NB conversion vs. reaction time given by different concentration of Cu-MOF-74.

In order to assess the influence of the catalyst concentration, a new reaction run was performed with DMF as solvent, at 120 °C, NB/Ph molar ratio of 2, two equivalents of K_2_CO_3_, and catalyst concentrations of 0, 1, 3, 5, and 7 mol % (Figure 5). Catalyst concentration was calculated based on the Cu/NB molar ratio values. A low NB conversion (ca. 10%) was achieved in the absence of solid catalyst (blank) (Figure 3). The presence of Cu-MOF-74 catalyst provided a remarkable initial rate in a mere 5 min of reaction, with values of NB conversion from 58% (1 mol % catalyst concentration) to 81% (7 mol % of catalyst concentration). After 2 h of reaction time and catalyst concentrations of 1 and 3 mol %, yields of NB conversion were 84% and 94%, respectively. An increase of catalyst concentration up to 5% led to 100% conversion in 90 min, but interestingly, an increase beyond 5% did not produce any significant improvement. Similar results were reported by Chen et al. for NB conversion when using 5 mol % of (Cu(OAc)_2_·H_2_O) as a catalyst and similar reaction conditions [12]. However, it must be pointed out that Cu content for reaching a complete conversion of NB as shown before is significantly lower compared to conventional Ullman reactions, where an average value of 10 mol % Cu-catalyst concentration is needed [27,28].

#### 2.2.4. Influence of the Solvent

**Figure 6:**
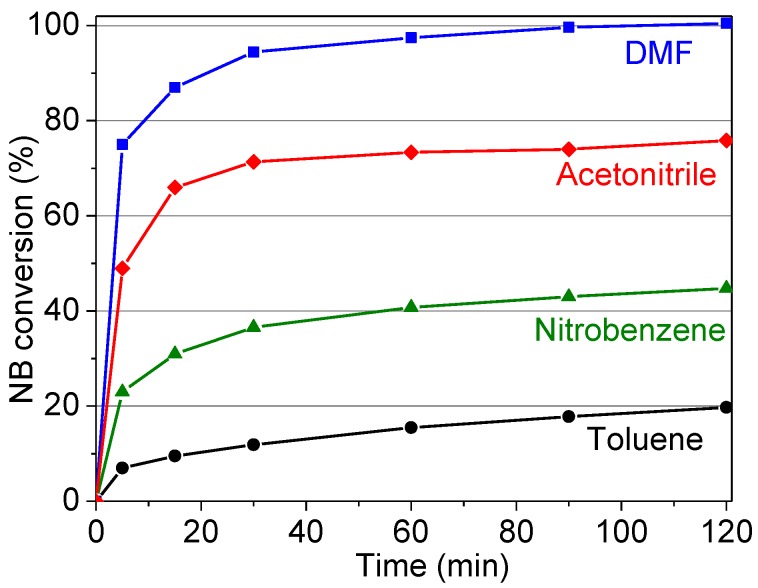
NB conversion vs. reaction time given by Cu-MOF-74 catalysts in different solvents.

The type of solvent can be crucial in coupling Ullmann reactions due to its influence on the reaction mechanism and solubility of reactants. Therefore, the O-arylation reaction of Ph with NB was carried out in different solvents as DMF, nitrobenzene, toluene, and acetonitrile, keeping constant the temperature (120 °C), the NB/Ph molar ratio (1/2), 2 equivalents of K_2_CO_3_ as a base, and 5 mol % of catalyst concentration. As shown in Figure 6, toluene was the worst solvent, with only 20% of NB conversion after 2 h of reaction. Under the same conditions, nitrobenzene also showed a relatively low conversion (around 43%), whereas acetonitrile led to a much higher conversion (75%), although still lower than the 100% conversion attained with DMF in hardly 1.5 h of reaction. These results are in good agreement with the literature using other MOF materials [20], homogeneous catalysts [12] and different Ullman reactions [4,29,30]. The solvent has also an important effect on homogeneous Ullmann O-arylation reactions of phenol with aryl chlorides using copper bromide in the presence of 1,10-phenantroline as ancillary ligand (L) [31]. In this case, the halogen atom transfer-based mechanism is sometimes proposed, but others based on oxidative addition, σ-bond, methathesis, or single electron transfer mechanisms have also been suggested. Hartwig et al. [32] described an equilibrium between neutral LCu–OAr (Cu(I)-phenoxide complex as a consequence of the base neutralization) and ionic forms of the Cu(L)_2_^+^ cation and Cu(OAr)_2_^−^ anion, as a result of disproportionation of LCu–OAr species. This equilibrium is controlled by the solvent polarity. Thus, the use of polar solvents favors the formation of ionic complexes, suggesting that ionic forms lead to the halogen atom transfer of aryl chlorides for the formation of diaryl ethers [33].

In the case of the O-arylation of phenol with nitrobenzaldehyde instead of aryl chlorides, this mechanism can be also responsible for the formation of ionic species over the unsaturated copper metal sites of the Cu-MOF-74, which promotes the coupling reaction with NB. Besides, it is also very important to point out the solvent influence on the solubility of reactants, based on its polarity. The solubility of NB in the reaction conditions ranged from 0.5 to 1.7 M for toluene and DMF, respectively. These results are in agreement with their polarity indexes, ranging from 2.3 to 6.4 for toluene and DMF, respectively. It is thus concluded that the effect of solvent on the solubility of NB could also be affecting the catalytic performance of the heterogeneous Cu-MOF-74 catalyst [31].

#### 2.2.5. Influence of the Base

**Figure 7:**
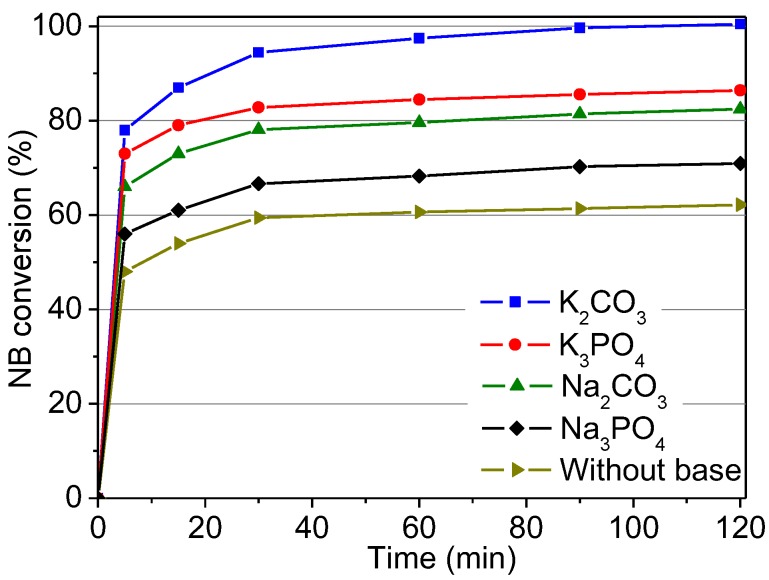
NB conversion vs. reaction time given by Cu-MOF-74 catalysts in the absence of base and in the presence of different bases.

The influence of the base on the catalytic activity of Cu-MOF-74 was also studied. Chen and co-workers proved that Cs_2_CO_3_ is more efficient than other bases for the coupling reaction between phenol and 4-nitrobenzaldehyde. However, due to the relatively high cost of Cs_2_CO_3_, other more readily available and inexpensive bases such as K_2_CO_3_, Na_2_CO_3_, K_3_PO_4_, and Na_3_PO_4_ were tested under the usual conditions: DMF as solvent, 120 °C, NB/Ph molar ratio of 2, two equivalent of base, and 5 mol % of catalyst. Figure 7 shows the NB conversion obtained for the different bases. An additional blank experiment with no base is also included. As observed, a significant NB conversion of 60% was achieved in the absence of base, but much higher values were obtained when the base was present. The catalytic activity of Cu-MOF-74 is quite influenced by the type of basic anion (carbonate or phosphate) and the counter-cation. Carbonates evidenced a better catalytic performance than phosphates for a given cation (either sodium or potassium). The higher kinetic diameter of phosphate anions is probably affecting the catalytic process of deprotonation of phenol inside the porous structure of the Cu-MOF-74 framework. Additionally, the use of potassium instead of sodium salts also showed a remarkable enhancement of the catalytic performance of Cu-MOF-74. This effect is attributed to the lower electronegativity of potassium cations. A higher electronegativity promotes a stronger affinity to deprotonated phenoxide anions, which would hinder the formation of Cu–phenoxide complexes and consequently the following transmetallation step for the formation of the diaryl ether compound.

**Figure 8:**
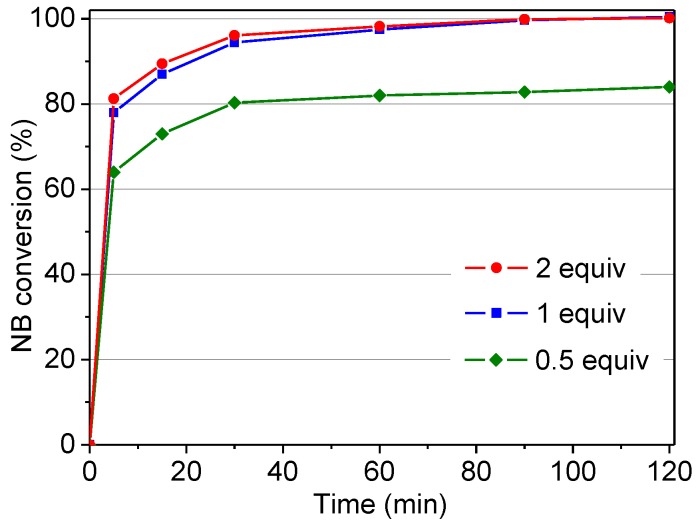
NB conversion vs. reaction time given by Cu-MOF-74 catalysts at different K_2_CO_3_ concentrations.

The influence of the K_2_CO_3_ base concentration was also studied by varying the base concentration from 1 to 0.5 and 2 equivalents. Figure 8 shows the NB conversion for the three catalytic runs in the usual conditions (DMF, 120 °C, NB/Ph = 2 and 5 mol % catalyst). The increase of the K_2_CO_3_ concentration from 0.5 to 1 equivalents enhances the catalytic activity of Cu-MOF-74. However, the increase up to 2 equivalents does not produce further conversion improvement, revealing that one equivalent (base/Ph molar ratio of 0.5) is able to promote an efficient deprotonation of phenol for the formation of phenoxide complexes with the Cu-containing sites of Cu-MOF-74 material.

#### 2.2.6. Influence of Different Substrates

**Figure 9:**
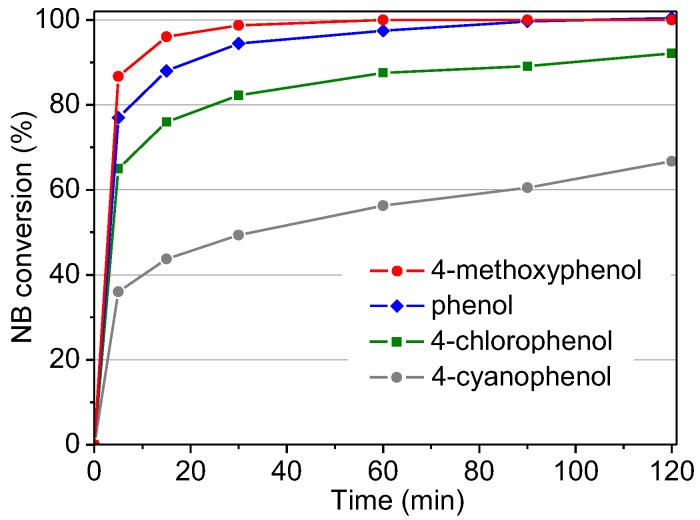
NB conversion vs. reaction time using different substituted-phenols.

The catalytic activity of Cu-MOF-74 material was also evaluated for different nitroarenes and phenol derivatives. The reactions were performed using DMF as solvent, at 120 °C, nitroarene/Ph molar ratio of 1/2 and 1 equivalent of K_2_CO_3_. Figure 9 shows the NB conversions when 4-cyanophenol, 4-chlorophenol, 4-methoxyphenol, and Ph were used. The coupling reaction was accelerated for the phenol derivative with electron-donating groups such as 4-methoxyphenol, attaining 87% of NB conversion in a mere 5 min. Phenol was slightly less active at early times than 4-methoxyphenol, but in both cases affording 100% after 90 min. The presence of electron-withdrawing groups in phenol derivative, such as the cases of 4-chlorophenol and 4-cyanophenol, decreased the NB conversion significantly.

**Figure 10:**
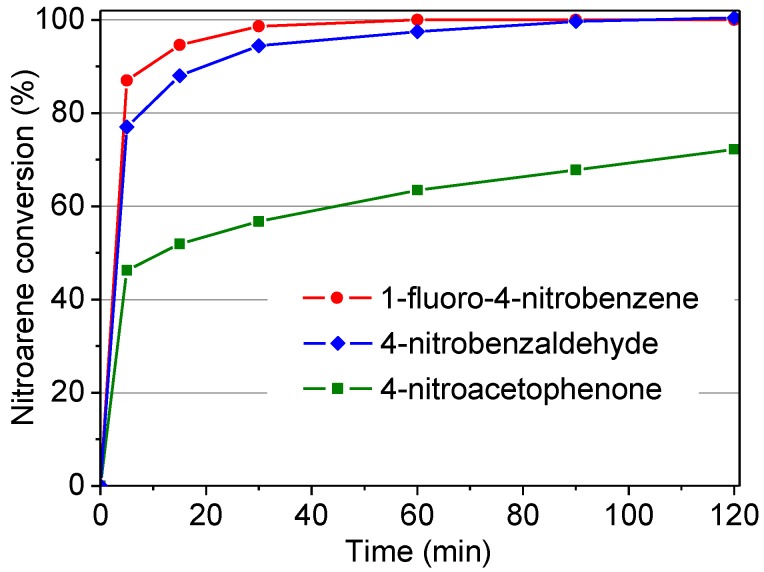
NB conversion vs. reaction time using different nitroarenes.

Figure 10 showed the conversion of different nitroarenes compounds when 1-fluoro-4-nitrobenzene, 4-nitroacetophenone, and 4-nitrobenzaldehyde were used in the presence of phenol. The highest conversion was obtained for 1-fluoro-4-nitrobenzene affording almost 100% in only 30 min, followed by 4-nitrobenzaldehyde (100% in 90 min) and finally 4-nitroacetophenone (72% in 120 min). This fact suggests that nitroarene compounds with electron-withdrawing groups, such as the case of 1-fluoro-4-nitrobenzene, allow for an enhancement in the catalytic performance. In contrast, electron-donating groups have shown the opposite effect, as has also been observed by other authors [12,20]. These results are in agreement with those published by Phan et al. for this reaction using a copper-based MOF catalyst (Cu_2_(BDC)_2_(DABCO)) [20]. However, Cu-MOF-74 evidenced a lower dependence on the electron-donating/withdrawing character of the substituents of phenol and nitroarenes in the overall catalytic performance.

#### 2.2.7. Comparison with Other Cu-Catalysts

**Figure 11:**
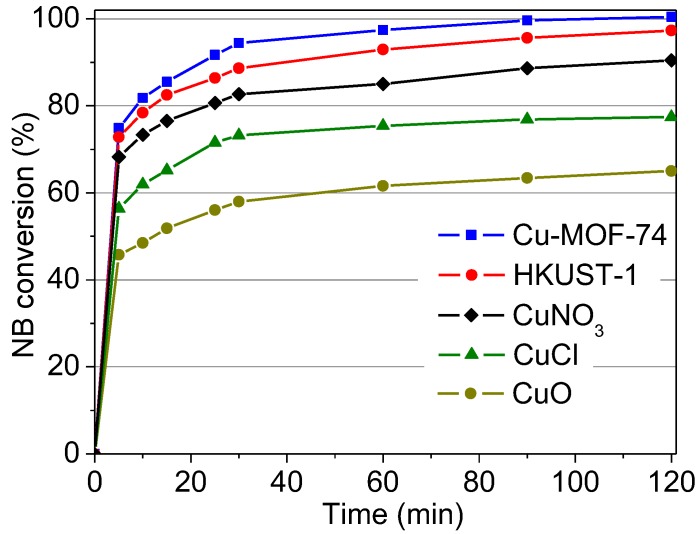
NB conversion vs. reaction time given by different homogeneous and heterogeneous Cu-based catalysts.

The catalytic performance of Cu-MOF-74 in the cross-coupling reaction of phenol with 4-nitrobenzaldehyde was also compared with other Cu-based heterogeneous catalysts (CuO and HKUST-1) and homogeneous catalysts (CuCl and CuNO_3_ salts). The catalyst concentration was always set to copper contents of 5 mol %. The experiments were carried out using DMF as solvent, at 120 °C, an NB/Ph molar ratio of 1/2 and 1 equivalent of K_2_CO_3_. Figure 11 shows the best catalytic performance of Cu-MOF-74 (100% conversion at 2 h) compared to the other Cu-materials. The homogeneous catalytic systems based on CuCl and CuNO_3_ salts still show high NB conversions after 2 h, albeit below 100%. This decrease in activity was attributed to possible negative effects of Cl^−^ and NO_3_^−^ anions in the O-arylation cross-coupling reaction. This was confirmed by additional experiments performed with an extra amount (1.165 mg/mL) of Cl^−^ and NO_3_^−^, provided by potassium-based salts. These experiments showed a significant decrease in NB conversion to 62% and 76% for CuCl and CuNO_3_, respectively, demonstrating the strong interference of the anions in the reaction course. On the other hand, CuO catalyst showed the worst catalytic performance (65% conversion), indicating the crucial role of the microporous structure of Cu-MOF-74, which provides a higher availability of copper sites compared to the very low porosity of CuO (34 m^2^/g). In the case of HKUST-1 (a well-known Cu-based MOF material also containing open metal sites), the catalytic performance was slightly lower than for Cu-MOF-74. The lower activity is probably due to the higher specific surface area of Cu-MOF-74 (1126 m^2^/g) compared to that of HKUST-1 (708 m^2^/g) and the better accessibility to the open metal sites of MOF-74 structure.

#### 2.2.8. Effect of Nanostructured Cu-MOF-74

**Figure 12:**
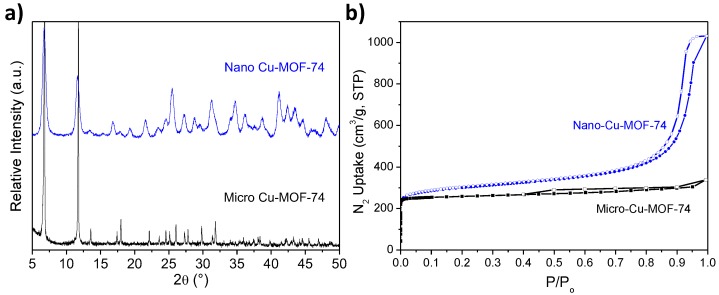
(**a**) PXRD patterns; (**b**) nitrogen adsorption–desorption isotherms at −196 °C of nano sized- and micron sized-Cu-MOF-74.

With the purpose of studying the effect of the crystal size over the catalytic performance of Cu-MOF-74 material, nanostructured homologue was also tested in this reaction and compared to the solvothermally synthesized microcrystalline material under the same reaction conditions. Nanocrystalline Cu-MOF-74 was prepared as described elsewhere [34] following a general method for synthesizing different M-MOF-74 materials at room temperature [35]. XRD powder patterns of nano- and micro-Cu-MOF-74 are depicted in Figure 12a. Both samples displayed the most important reflexions of Cu-MOF-74 crystalline phase, the nanomaterial being obviously broader than the ones of the conventional micro-sized MOF material. N_2_ adsorption/desorption isotherms at −196 °C are shown in Figure 12b and were used for estimating the textural properties of these Cu-MOF-74 samples (Table 1).

**Figure 13:**
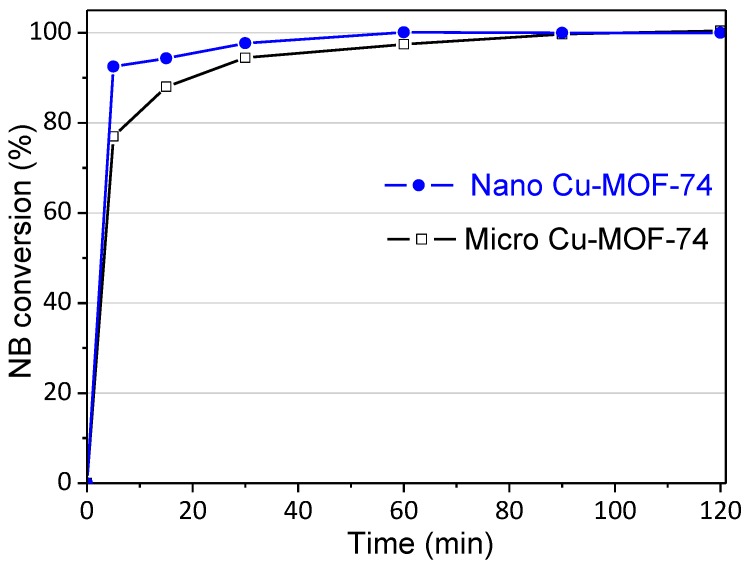
NB conversion vs. reaction time given by nano- and micron-sized Cu-based catalysts.

Regarding the catalytic results depicted in Figure 13, nanostructured Cu-MOF-74 showed faster reaction kinetics than its microcrystalline homologue, reaching nitrobenzaldehyde conversions of 93% and 76%, respectively, after 5 min of reaction. This higher NB conversion at early times using nanoparticles can be attributed to its more than double external surface area compared to the micro-Cu-MOF-74, showing an extra amount of readily accessible copper active sites. These promising results demonstrated that, using nanocrystalline catalysts, the reaction system can exhibit the main advantages of both strategies: highly active homogeneous catalysis as well as eco-friendly heterogeneous catalysis. In good agreement with our results, the advantages of using nanocrystalline catalysts against their counterparts formed by micron-sized crystals have been already evidenced for some other MOF-based catalysts [36] and even for these based on M-MOF-74 [34]. 

#### 2.2.9. Reuse of Cu-MOF-74

**Figure 14:**
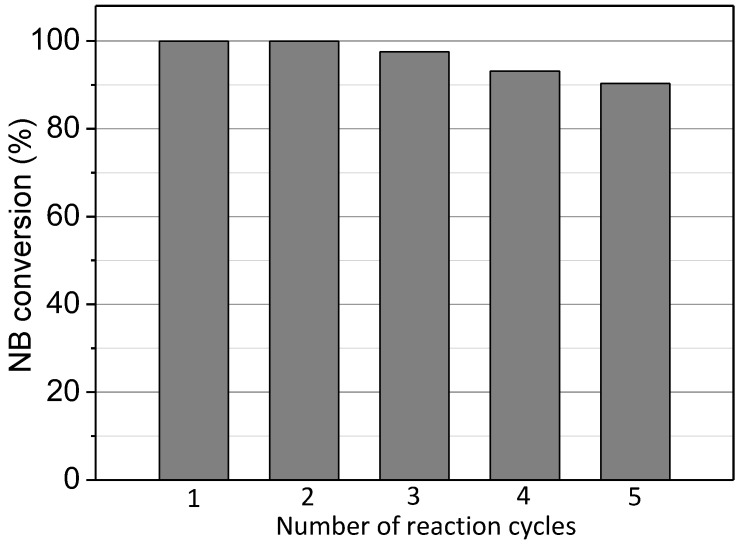
Catalytic activity of Cu-MOF-74 catalyst after several reaction cycles.

**Figure 15:**
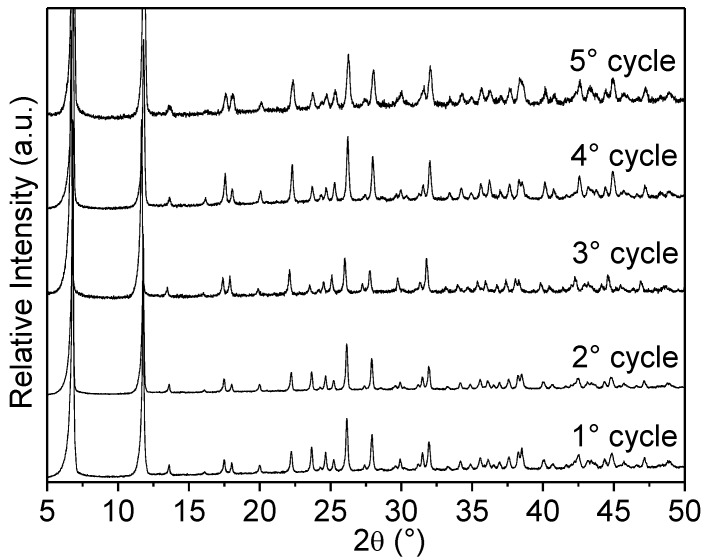
XRD patterns of Cu-MOF-74 catalyst after several reaction cycles.

An important feature of chemical processes based on organic reactions from the environmental point of view is the reusability of the catalyst in a number of reaction cycles. Therefore, the recyclability of the catalyst was also studied in the O-arylation cross-coupling reaction for five successive catalytic runs. Prior to each catalytic run, the catalyst was recovered and washed several times with methanol and dried at 150 °C for 18 h. The NB conversion and the XRD patterns of the catalyst along the five cycles are shown in Figure 14 and Figure 15, respectively. The catalytic performance was above 90% after the five cycles, although a slight decrease in activity was observed after the third one (Figure 14). XRD measurements of Figure 15 show that the crystalline phase of Cu-MOF-74 keeps unaltered, meaning that the catalyst structure is stable. The copper leaching was negligible, as commented above, and the catalyst recovery after each cycle was above 97%. All these results demonstrate that the crystalline framework of Cu-MOF-74 material is stable under the tested reaction conditions, the slight deactivation observed possibly being due to chemisorbed by-products along the reaction cycles that were not completely removed during the regeneration process.

## 3. Materials and Methods 

### 3.1. Catalysts Preparation

Cu-MOF-74 was synthesized basically following the procedure previously published [24], in which a mixture of 2.2 g of 2,5-dihydroxyterephthalic acid (H_2_dhtp, 11.2 mmol, Sigma-Aldrich, Madrid, Spain) and trihydrated copper nitrate(II) (5.9 g, 24.6 mmol, Sigma-Aldrich, Madrid, Spain) were added to a 250 mL solution of *N*,*N*-dimethylformamide (DMF) and 2-propanol (20:1 (*v*/*v*)) in a 500 mL screw cap bottle. The suspension was stirred until a homogeneous solution. Then, the resultant solution was placed in an oven at 80 °C for 18 h. Thereafter, the sample was cooled down to room temperature and the mother liquor was separated from reddish needle-shaped crystals by vacuum filtration. Afterwards, the crystalline solid sample was washed with DMF and immersed in 100 mL of methanol for 4 days, renewing it by fresh methanol every 24 h. Prior to the catalytic runs, the solid material was dried at 150 °C under vacuum (10^−3^ bar) for 5 h and stored under inert atmosphere. Other powdered materials used in this study with the purpose of comparison were HKUST-1 (Basolite C300, supplied by Sigma-Aldrich, Madrid, Spain) as a different copper-based MOF material and CuO. Likewise, homogeneous copper salts such as CuCl_2_ and Cu(NO_3_)_2_ were used as homogeneous catalysts (Sigma-Aldrich, Madrid, Spain). Nanocrystalline Cu-MOF-74 was prepared at room temperature as described elsewhere [34]. 

### 3.2. Catalyst Characterization

X-ray powder diffraction (XRD) patterns were acquired on a PHILIPS X’PERT diffractometer using Cu Kα radiation. The data were recorded from 5° to 50° (2θ) with a resolution of 0.01°. Scanning electron microscopy (SEM) micrographs were obtained on a XL30 ESEM (Philips, Lelyweg, the Netherlands) electronic microscope operating at 200 kV. Nitrogen adsorption–desorption isotherms at −196 °C were measured using an AutoSorb equipment (Quantachrome Instruments, Boynton Beach, Florida, USA). Samples were degassed at 150 °C and high vacuum during 180 h. The micropore surface area was calculated by using the Brunauer–Emmett–Teller (BET) model [37]. The pore volume and diameter were estimated by non-local DFT calculations, assuming a kernel model of N_2_ at −196 °C on carbon (cylindrical pores, NL-DFT equilibrium model) [38] and external surface was estimated by t-plot method and Harkins Jura equation [39]. Simultaneous thermogravimetry and derivative thermogravimetric analyses (TGA/DTG) were carried out under a nitrogen atmosphere with an N_2_ flow of 100 mL·min^−1^ at a heating rate of 5 °C/min up to 700 °C, using a SDT 2860 apparatus (TA Instruments, New Castle, DE, USA).

### 3.3. Reaction Procedure

**Scheme 1:**
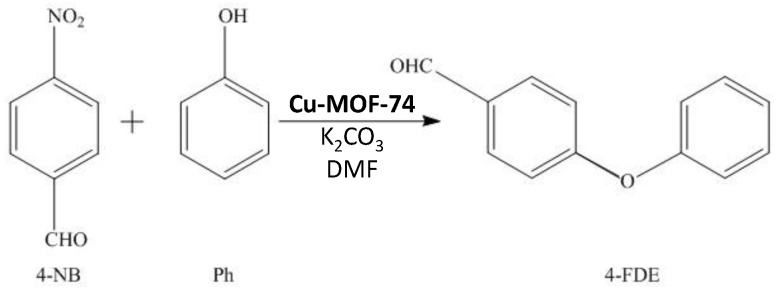
C–O cross-coupling reaction of phenol with 4-nitrobenzaldehyde.

Cu-MOF-74 material was tested in the O-arylation cross coupling reaction of phenol (Ph) and 4-nitrobenzaldehyde (NB) to form 4-formyldiphenyl ether (FDE) (Scheme 1). All the catalytic experiments were carried out in a round bottom flask placed in a silicone bath under N_2_ atmosphere. The influence of the reaction temperature, the molar ratio of reactants (NB/Ph), the catalyst concentration, the type of solvent and both base nature and concentration, were evaluated according to preliminary conditions found in the literature [20]. The required amounts of reactants (Ph and NB) were added to 20 mL of the solvent. The base and catalyst concentrations were adjusted according to molar ratios of base/Ph and Cu/NB, respectively. The reaction was monitored by withdrawing aliquots from the reaction medium at different times ranging from 0 to 120 min. The NB and FDE were identified and quantified by gas chromatography, using a GC-3900 chromatograph with a flame ionization detector (FID) (Varian, Palo Alto, CA, USA). A CPSIL 8 CB capillary column was used as stationary phase of 30 m × 0.25 mm andfilm thickness of 0.25 µm (Agilent Technologies Spain, Madrid, Spain). The injector and FID temperatures were set to 280 °C, and the oven temperature program started at 120 °C for 1 min and continued until 280 °C at 40 °C/min^−1^ and 3 min at 280 °C. Hexadecane was used as an internal standard, and all samples were analyzed in duplicate. 

The relative NB conversion was calculated taking into account the maximum theoretical NB conversion, which depends on the 4-NB/Ph ratio of each catalytic run. The selectivity to diaryl ether FDE was considered of 100% as no other by-products were detected. A similar assumption was observed in the literature for this reaction [20]. 

## 4. Conclusions

Outstanding catalytic activity of Cu-MOF-74 in the O-arylation cross-coupling reaction of 4-nitrobenzaldehyde (NB) and phenol has been evidenced. A catalyst concentration of 5 mol % exhibits a total conversion of NB and 100% selectivity to the desired product after 2 h at 120 °C, whereas only 10% is achieved in the absence of catalyst. This O-arylation reaction only proceeds in the presence of Cu-MOF-74 catalyst, discarding a possible contribution of homogeneous catalysis due to active leached species in the liquid phase. The excess of phenol as well as a high polarity of the solvent plays an important role in the catalytic performance of Cu-MOF-74. The best conditions for the reaction are NB/Ph ratio of 1/2, DMF as solvent, and an inexpensive K_2_CO_3_ base. The catalytic performance of Cu-MOF-74 was dependent on the electron-donating/withdrawing character of the groups of phenol derivatives and nitroarenes. The catalytic activity of Cu-MOF-74 (above 90%) is higher than those shown by other homogeneous and heterogeneous Cu-based catalysts, and the material is stable and reusable during several reaction cycles. Nano Cu-MOF-74 catalyst exhibited faster reaction kinetics than its microcrystalline homologue, demonstrating the advantages of increasing the easy and quick accessibility of the reactants to the active centres. 

## Figures and Tables

**Table 1:** Textural properties of nano- and micro-Cu-MOF-74 materials.

Cu-MOF-74	Crystal Size (nm)	S_BET_ (m^2^/g)	S_External_^c^ (m^2^/g)
Nanocrystalline	14 ^a^	1103	286
Microcrystalline	10,000 ^b^	1126	124

^a^ Estimated by Scherrer equation. ^b^ Estimated by SEM. ^c^ Estimated by t-plot method.
